# A phase I/II study of bevacizumab, irinotecan and erlotinib in children with progressive diffuse intrinsic pontine glioma

**DOI:** 10.1007/s11060-021-03763-1

**Published:** 2021-05-07

**Authors:** Fatma E. El-Khouly, Sophie E. M. Veldhuijzen van Zanten, Marc H. A. Jansen, Dewi P. Bakker, Esther Sanchez Aliaga, N. Harry Hendrikse, W. Peter Vandertop, Dannis G. van Vuurden, Gertjan J. L. Kaspers

**Affiliations:** 1 Emma Children’s Hospital, Amsterdam UMC, Vrije Universiteit Amsterdam, Department of Pediatric Oncology, Amsterdam, The Netherlands; 2 Princess Máxima Center for Pediatric Oncology, Utrecht, The Netherlands; 3 Erasmus MC, Department of Radiology & Nuclear Medicine, Rotterdam, The Netherlands; 4 Wilhelmina Children’s Hospital, Department of Immunology, Utrecht, The Netherlands; 5 Emma Children’s Hospital, Amsterdam UMC, Vrije Universiteit Amsterdam, Department of Child Neurology, Amsterdam, The Netherlands; 6 Amsterdam UMC, Vrije Universiteit Amsterdam, Department of Radiology & Nuclear Medicine, Amsterdam, The Netherlands; 7 Amsterdam UMC, Vrije Universiteit Amsterdam, Department of Clinical Pharmacology & Pharmacy, Amsterdam, The Netherlands; 8 Amsterdam UMC, Neurosurgical Center Amsterdam, Amsterdam, The Netherlands

**Keywords:** Diffuse intrinsic pontine glioma (DIPG), Targeted therapy, Bevacizumab, Irinotecan, Erlotinib

## Abstract

**Introduction:**

This study investigates the safety, tolerability, and preliminary efficacy of combined treatment with VEGF inhibitor bevacizumab, topoisomerase I inhibitor irinotecan, and EGFR inhibitor erlotinib in children with progressive diffuse intrinsic pontine glioma (DIPG).

**Methods:**

Biweekly bevacizumab (10 mg/kg) and irinotecan (125 mg/m^2^) were combined with daily erlotinib. Two cohorts received increasing doses of erlotinib (65 and 85 mg/m^2^) following a 3 + 3 dose-escalation schedule, until disease progression with a maximum of one year. Dose-limiting toxicities (DLT) were monitored biweekly. Secondary progression free survival (sPFS) and overall survival (OS) were determined based on clinical and radiological response measurements. Quality of life (QoL) during treatment was also assessed.

**Results:**

Between November 2011 and March 2018, nine patients with disease progression after initial radiotherapy were enrolled. Median PFS at start of the study was 7.3 months (range 3.5–10.0). In the first dose cohort, one patient experienced a DLT (grade III acute diarrhea), resulting in enrollment of three additional patients in this cohort. No additional DLTs were observed in consecutive patients receiving up to a maximum dose of 85 mg/m^2^. Median sPFS was 3.2 months (range 1.0–10.9), and median OS was 13.8 months (range 9.3–33.0). Overall QoL was stable during treatment.

**Conclusions:**

Daily erlotinib is safe and well tolerated in doses up to 85 mg/m^2^ when combined with biweekly bevacizumab and irinotecan in children with progressive DIPG. Median OS of the study patients was longer than known form literature.

**Supplementary Information:**

The online version contains supplementary material available at 10.1007/s11060-021-03763-1.

## Introduction

Patients suffering from diffuse intrinsic pontine glioma (DIPG) face a dismal prognosis, with a median overall survival of eleven months and a two-year survival rate of 10% [1]. Radiotherapy remains the only, temporary, effective treatment and confers a survival benefit of approximately three months [2, 3]. Thus far, chemotherapy has not proven to be effective, either at diagnosis or at disease progression [3–5]. In the 2016 World Health Organization classification of central nervous system tumors, DIPGs were reclassified as Diffuse Midline Gliomas with a H3K27M-mutation [6].

Targeting multiple pathways has been stated to reduce the risk of drug resistance [7, 8]. Combining the humanized anti-VEGF monoclonal IgG_1_ antibody bevacizumab with the topoisomerase-I inhibitor irinotecan showed significant response rates in adult glioblastoma patients [9, 10]. The combination of bevacizumab (10 mg/kg) and irinotecan (125 mg/m^2^), has also been demonstrated to be safe and well tolerated in children with recurrent low- and high-grade glioma, including DIPG [11, 12].

In pediatric high-grade glioma (HGG) and DIPG, overexpression of EGFR has been consistently demonstrated [13–15]. Erlotinib, an EGFR tyrosine-kinase-inhibitor, blocks activation of EGFR by reducing its ability to phosphorylate substrates and in turn affects intracellular pathways through signal transduction [16]. In children with refractory solid tumors, erlotinib was safe and well tolerated up to 120 mg/m^2^ [17, 18].

Based on their mechanism of action, targeting different pathways, adding erlotinib to a backbone therapy of bevacizumab and irinotecan could provide a larger inhibitory effect on tumor proliferation. Moreover, binding VEGF by bevacizumab also lowers the interstitial pressure and increases vascular permeability. This may increase delivery of systemic chemotherapeutic agents like irinotecan and erlotinib, possibly enhancing their potential [19]. In this study we aimed to (i) determine safety and tolerability of adding erlotinib to a backbone therapy of bevacizumab and irinotecan, (ii) determine preliminary efficacy in terms of secondary progression free survival (sPFS) and overall survival (OS), and (ii) evaluate quality of life (QoL) during treatment.

## Methods

### Approval

This study is part of a larger two-phased clinical trial “A comprehensive and targeted therapy approach in pediatric malignant pontine gliomas” (EudraCT 2009-016080-11, Dutch Trial Register NTR2391), approved by the ethical committee of Amsterdam UMC, location VUmc (study number: VUMC2010/164), and the Scientific Committee of the Dutch Childhood Oncology Group. The first phase of this trial was a phase I/II, open-label, single-arm trial investigating the safety, tolerability, and preliminary efficacy of gemcitabine as a radiosensitizer, administered concomitantly to radiotherapy in newly-diagnosed DIPG patients [20]. For this second phase, separate informed consent was obtained from all parents of children participating in the trial, and informed assent was obtained from patients aged 12–18 years. All study procedures took place at Amsterdam UMC, location VUmc, in Amsterdam, the Netherlands.

### Premature ending of the trial

Halfway during the trial, pediatric oncology care in the Netherlands was centralized in a new dedicated pediatric oncology hospital known as the Princess Máxima Center in Utrecht. Due to an initial slow inclusion rate in a non-centralized setting at the Amsterdam UMC, location VUmc and later logistic difficulties transferring the trial to the Princess Máxima Center, this study had to be terminated prematurely before we could escalate to the final dose cohort(s) prescribing also everolimus (in escalating doses of 2 mg/m^2^ and 3 mg/m^2^, respectively).

### In- and exclusion criteria

Children aged 3–18 years with progressive DIPG were eligible for this study. The following patients were eligible for inclusion: (i) patients with clinical or radiological disease progression after initial therapy, (ii) patients who participated in the first phase of this trial experiencing progressive disease, (iii) patients with progressive disease who did not participate in the first phase of this trial but underwent at least radiotherapy (conventional or hypo-fractionated) at diagnosis, (iv) written informed consent, (v) transfusion-independent platelet count ≥ 75 × 10^9^/L, (vi) peripheral absolute neutrophil count (ANC) ≥ 0.75 × 10^9^/L, (vii) adequate liver function, defined as direct bilirubin ≤ 1.5 × upper limit of normal (ULN) for age and alanine aminotransferase (ALAT) < 5 × upper limit of normal (ULN) for age, (viii) adequate renal function, defined as serum creatinine ≤ 1.5 × upper limit of normal (ULN) for age, (ix) willingness to perform a pregnancy test and apply contraceptives in females of child-bearing age. Biopsy was offered as an option, but was not mandatory. Exclusion criteria were: (i) patients who received radiotherapy or chemotherapy in the past 2 weeks, (ii) pregnant or breastfeeding, (iii) contra-indications for chemotherapy or targeted therapy, (iv) clinically-diagnosed neurofibromatosis type I (DNA-diagnostics not mandatory), (v) performance status (Lansky or Karnofsky score) of ≤ 40.

### Study objectives and definitions

The primary objective of this study was to determine safety and tolerability of adding erlotinib, in two pre-specified dose-levels, to a backbone therapy of bevacizumab and irinotecan. The secondary objective was to evaluate preliminary efficacy in terms of sPFS and OS. Clinical disease progression was defined as neurological deterioration compared to baseline (i.e. worsening of existing or emergence of new symptoms). Radiological progression was defined based on the modified RANO criteria as either tumor growth or leptomeningeal metastasis after radiotherapy as determined by the neuro-radiologist [21]. Secondary progression was defined as significant increase of symptoms or development of new symptoms and/or radiological progression after initiation of the study. The tertiary objective was to evaluate QoL during therapy using the Quality of Life Inventory™ (PedsQL) questionnaires.

### Study procedures

All patients received a central venous catheter (port-a-cath or Broviac) in view of the intensity and duration of systemic therapy. Patients received chemotherapy in 2-weeks during courses for a maximum period of one year (26 courses). The backbone therapy, consisting of bevacizumab 10 mg/kg and irinotecan 125 mg/m^2^, was administered intravenously every 2 weeks. Two successive cohorts received escalating doses of erlotinib (65 mg/m^2^ and 85 mg/m^2^ once daily, orally). Doses were escalated following a 3+3 dose-escalation schedule meaning that if a dose limiting toxicity (DLT) was observed in one out of three patients in a specific cohort, three additional patients would be enrolled in that cohort [22]. The maximum-tolerated dose (MTD) would be reached if more than one out of six patients, in one cohort, developed a DLT (i.e. grade ≥ 3 adverse event). In that case, further dose-escalation of erlotinib would not be pursued. If no DLT was observed in a specific cohort at 2 weeks after erlotinib administration, additional patients were treated following the next dose-level of 85mg/m^2^. After establishing the MTD of erlotinib, patients in the following cohorts were initially planned to also receive escalating doses of everolimus (2 mg/m^2^ and 3 mg/m^2^) added to the combination of bevacizumab, irinotecan, erlotinib, again following a 3 + 3 dose-escalation schedule. However, due to premature termination of this study, no patients were included in these cohorts.

Bevacizumab and irinotecan were reconstituted in 0.9% sodium chloride solution and administered intravenously via central access. The initial infusion time of bevacizumab was 90 minutes. When no allergic reaction occurred following the first administration, bevacizumab was infused in 60 minutes the second administration and, when tolerated, in 30 minutes at subsequent infusions. Prior to bevacizumab administration, irinotecan was administered in 60 minutes. Erlotinib was available in tablets containing 25 mg, 100 mg and 150 mg. Tablets were taken orally in the morning, at least one hour before or two hours after breakfast.

Prior to each cycle, patients were required to qualify based on hematological examination: ANC ≥ 0.75 × 10^9^/L, and platelet count ≥ 75 × 10^9^/L.

### Safety assessments and response evaluation

We assessed safety (i.e. evaluation of DLTs) during the first two treatment courses (i.e. over the first 4 weeks of the total treatment period). A DLT was defined as any clinically relevant, and likely drug-related, grade ≥ 3 adverse event, according to criteria outlined in the NCI Common Terminology Criteria for Adverse Events (CTCAE), version 4.03 [23]. We did not consider asymptomatic laboratory abnormalities a DLT.

Evaluation of DLTs included biweekly examination of complete hematological blood count (hemoglobin, platelets, white blood cell count and differentiation), serum chemistry (creatinine, blood urea, nitrogen, uric acid, albumin, sodium, potassium, calcium, magnesium, phosphate, ASAT, ALAT, γ-GT, bilirubin, LDH, bicarbonate, glucose), urine analysis to check for proteinuria and also measurement of blood pressure.

Patients additionally underwent biweekly physical and neurological examination by either a pediatric oncologist or a child neurologist in order to assess possible DLTs and disease progression. Following the first 4 weeks of the study, we performed extensive neurological examination monthly to assess possible efficacy or disease progression during treatment. MRI-scans of the brain and spinal cord were performed at baseline and every three months during treatment or earlier in case disease progression was suspected. MR-images were evaluated by a neuro-radiologist using the modified RANO-criteria to determine tumor growth and/or the presence of leptomeningeal metastasis [21]. An echocardiography (ECG) was made before start of the study and every three months to detect possible cardiotoxicity (which is a known side-effect of bevacizumab treatment).

QoL was assessed at baseline and every three months during treatment using three categories of the using PedsQL-questionnaires: (i) PedsQL™ 4.0 Generic Core Scales, addressing physical performance and psychosocial health, (ii) the PedsQL™ Multidimensional Fatigue Scale, addressing general fatigue, sleep rhythm and cognitive fatigue and (iii) the PedsQL™ 3.0 Cancer Module, addressing pain during treatment, nausea, fear of treatment and procedures, worrying about disease course, appearances and communication with other people. Each PedsQL category provides age-appropriate questionnaires that take approximately ten minutes [24, 25].

At the end of treatment, either as a result of completing 26 courses or due to disease progression, clinical follow-up was performed every three months to determine sPFS and/or OS.

### Supportive care

In case of repeated nausea, patients were treated with ondansetron either intravenously (10–15 mg/m^2^) or orally (5 mg/m^2^), up to a maximum of 8 mg per dose, three times a day. The use of dexamethasone was avoided whenever possible because of associated side-effects [26]. Late-onset diarrhea was treated at home with loperamide. In case of early-onset (acute) diarrhea, atropine (0.01 mg/kg, maximum of 0.4 mg/dose) was administered. When weight loss of more than 10% occurred, a weight-gaining program was started under supervision of a dietician.

### Statistics

Data were analyzed by descriptive statistics using IBM SPSS Statistics version 26. Secondary PFS and OS were determined using the Kaplan-Meier method. PFS and OS of the total study cohort were compared to historical survival data of DIPG patients. The DIPG survival-prediction model was used to determine the risk-category of each patient, to evaluate whether predictive factors could have influenced survival in this prospective treatment-study [2, 27]. Risk-scores were calculated based on three variables: (i) symptom duration (in months) at time of diagnosis, (ii) age at diagnosis, and (iii) presence of ring enhancement on diagnostic MRI. Based on the risk-scores, patients were categorized as either standard- (score ≤ 1), intermediate- (score 1–6) or high-risk (score ≥ 7). For each risk-group subgroup specific PFS and OS were calculated, and compared to the survival data reported by Jansen et al. [2].

## Results

### Patients

Between November 2011 and March 2018, nine patients with progressive DIPG were enrolled in this study. Four patients previously participated in the first phase of this trial at diagnosis and received radiotherapy combined with gemcitabine as radiosensitizer [20]. The other five patients were initially treated with radiotherapy only. According to the DIPG survival-prediction model, patients were classified as being intermediate- (*n* = 4) or high-risk (*n* = 5) at diagnosis with scores varying from 3.0–0.8 [2]. Median PFS after initial therapy was 7.3 months (range 3.5–10.0). Patients from whom either biopsy or autopsy tissue was available (four out of nine), harbored H3K27M mutation. Patient characteristics are summarized in Table 1.

**Table 1:** Baseline characteristics of DIPG patients

Patient ID	Gender	Age at diagnosis (y)	Histology	Risk group	Initial therapy	PFS, i.e. start study (mo)	study cohort
1	F	6.7	n.a.	High	RTx only	3.5	1
2	F	17.2	DMG H3K27M (WHO III)	High	RTx + chemo^A^	5.1	1
3	M	11.8	n.a.	High	RTx + chemo^A^	6.3	1
4	M	14.6	DMG H3K27M^a^ (WHO I-IV)	High	RTx only^a^	7.5	1
5	M	7.4	DMG H3K27M (WHO II)	Inter	RTx + chemo^B^	7.4	1
6	M	7.7	DMG H3K27M^a^ (WHO I-IV)	Inter	RTx + chemo^C^	10.0	1
7	F	9.7	n.a.	High	RTx only	8.4	2
8	M	5.9	n.a.	Inter	RTx only	7.3	2
9	F	5.2	n.a.	Inter	RTx only	6.0	2
Median		7.7				7.3	

*F* female, *M* male, *y* year, *n.a.* not applicable, no biopsy or autopsy performed, *High* high-risk patients, *Inter* intermediate-risk patients, *RTx only* radiotherapy 39 Gy (13 × 3 Gy), *RTx + Chemo* radiotherapy 54 Gy (30 × 1.8 Gy) + gemcitabine IV in doses of 140 mg/m^2^ (A), 175 mg/m^2^ (B), 200 mg/m^2^ (C)

^a^Radiotherapy 54 Gy (30 × 1.8 Gy)

### Toxicity

All patients received a combination of bevacizumab, irinotecan and erlotinib according to the predefined schedule. The first patient included in the first dose-cohort experienced grade II acute secretory diarrhea after the second cycle, treated with atropine. However, the diarrhea increased in the week after, up to 10 stools per day, which resulted in a grade III adverse event and thus a DLT. For this patient, irinotecan and erlotinib were stopped for 4 weeks. No diarrhea was reported after rechallenge. The occurrence of this DLT resulted in enrollment of three additional patients in that specific dose-cohort. In the following cohorts, five patients experienced grade I/II late onset diarrhea, which was treated with loperamide at home when necessary.

All patients experienced grade I/II nausea and vomiting on the day of administration of bevacizumab and irinotecan. Therefore, ondansetron was administered intravenously 15 minutes before irinotecan was started. In four out of nine patients, nausea and vomiting was also present two to three days after IV administration of bevacizumab and irinotecan for which oral ondansetron was prescribed. Nausea and vomiting disappeared directly after treatment was completed. Alopecia was observed in all patients and started after the third treatment course. Four out of nine patients experienced grade I acneiform rash in the form of papules and pustules around the nose, related to erlotinib. One patient experienced grade II acneiform rash with papules and pustules also covering the chest and back. Other observed adverse events were grade I/II mucositis (*n* = 1), grade I/II constipation (*n* = 1), grade II keratitis (*n* = 1), grade II urinary tract infection (*n* = 2), and grade II adrenal insufficiency as a result of chronic dexamethasone use (*n* = 2). Bevacizumab-related cardiotoxicity or proteinuria was not observed in any of the participating patients.

### Clinical/neurological response

At start of the study neurological symptoms such as ataxia, a positive Babinski reflex, facial nerve palsy, abducens nerve palsy and dysarthria were observed in all patients. Neurological symptoms were stable during the first three months after start of the study in four patients, and neurological progression was observed in five patients. When the disease progressed, additional symptoms, such as dysphagia, apathy, and abnormal gait or inability to walk were observed at secondary progression.

### Radiological response

At three months after start of treatment, partial radiological response was observed in three patients (patient two, four and eight, respectively), stable disease was observed in one patient (patient five), and progressive disease in five patients (patient one, three, six, seven, and nine, respectively) of whom one developed an intraventricular metastasis (patient seven). At 6 months, radiological response assessment showed progressive disease in two patients (patient four and five), and stable disease in two (patient two and eight) of whom one patient had clinical disease progression (patient eight) for which treatment was stopped. The last patient (patient two) showed radiologic progression after one year of treatment. No differences in radiologic responses were observed between dose-levels. Complete radiologic assessment can be found in supplementary Table 1.

### Survival

**Fig. 1:**
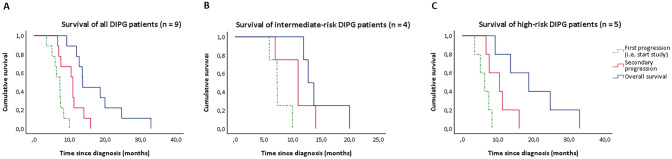
Cumulative survival of DIPG patients: first progression (PFS)/start of the study (green dotted line), secondary progression/progression after study treatment (red line), and overall survival (blue line) for all study patients (**a**), intermediate-risk patients (**b**), and high-risk patients (**c**)

**Fig. 2:**
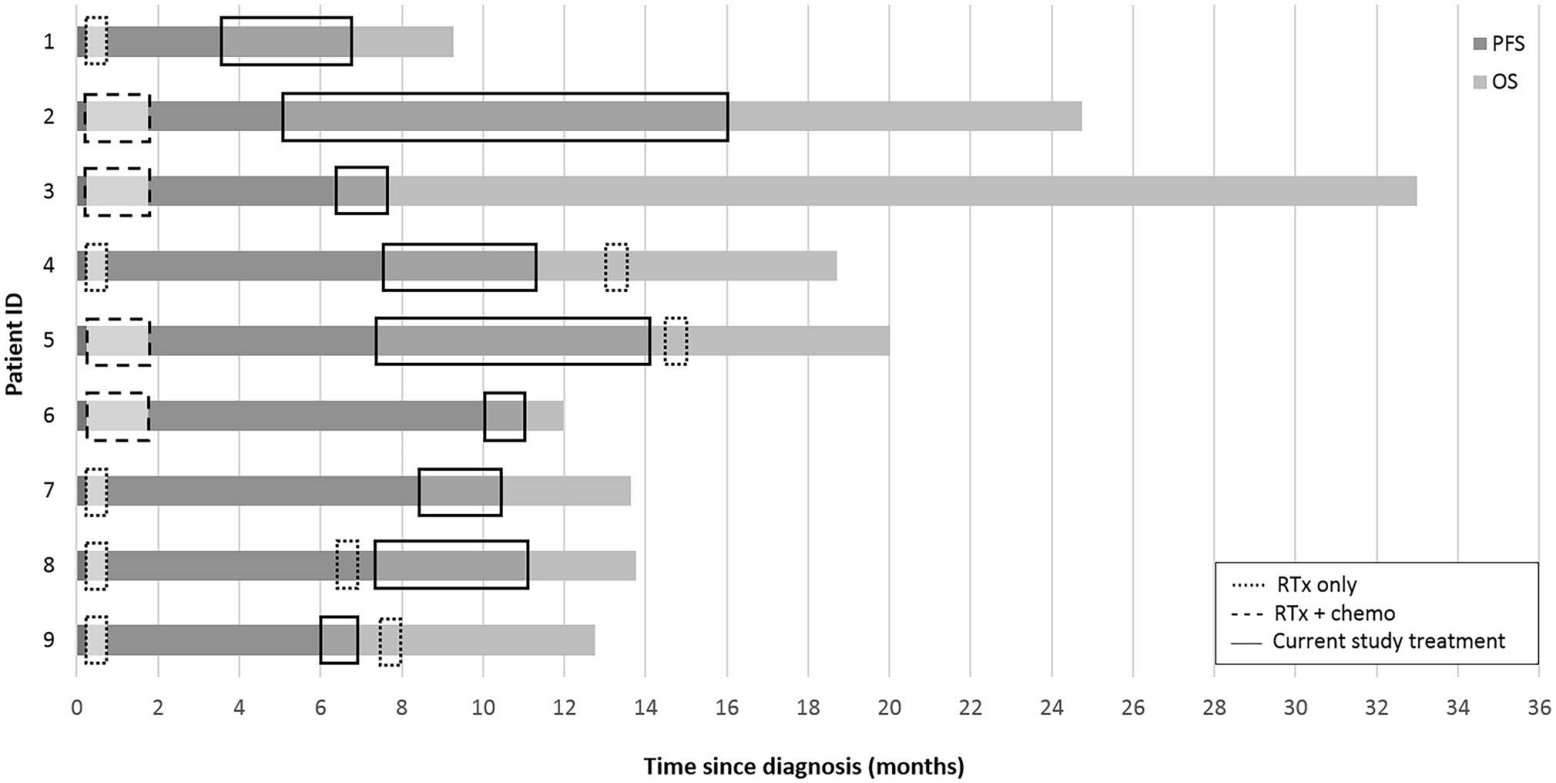
Disease course for every patient included in this study, from diagnosis until death. (PFS: progression free survival; OS: overall survival; RTx: radiotherapy; chemo: chemotherapy)

Median sPFS and OS of all nine patients was 3.2 months (range 1.0–10.9) and 13.8 months (range 9.3–33.0), respectively (Fig. 1a). No significant difference in survival was observed between different dose-levels. When stratified for risk-category, PFS, sPFS and OS of intermediate-risk patients (*n* = 4) was 7.3 months (range 6.0–10.0), 1.0 months (range 1.0–6.7) and 12.8 months (range 12.0–20.0), respectively (Fig. 1b). For high-risk patients (*n* = 5) PFS, sPFS and OS was 6.3 months (range 3.5–8.4), 3.2 months (range 1.3–10.9), and 18.7 months (range 9.3–24.7), respectively (Fig. 1c). Figure 2 provides an overview of the disease course per patient, including the treatments received at diagnosis and after secondary progression. The OS of patients that were re-irradiated (*n* = 4) was 16.2 months (range 12.8–20.0), versus 13.6 month (range 9.3–33.0) for patient who did not pursue further treatment (*n* = 5).

### Quality of Life

Only four patients and their parents filled in the QoL questionnaires at two or more time points. QoL in these patients was not significantly different between time points. Based on the questionnaires, a slight reduction in QoL was observed when considering physical performance, nausea and fear of procedures/treatments (data not shown).

## Discussion

This phase I/II open-label single arm study demonstrates that multi-targeted therapy with biweekly bevacizumab (10 mg/kg) and irinotecan (125 mg/m^2^) combined with daily erlotinib in doses up to 85mg/m^2^ is safe and well tolerated in children with DIPG at disease progression.

Although not powered on efficacy, we reported higher overall survival rates compared to survival data of DIPG patients receiving radiotherapy only, 13.8 months versus 10 months, respectively [5]. When comparing the data stratified according to risk-group, the median OS of our intermediate- and high-risk patients was significantly higher compared to the population used to develop the model, 12.8 months and 18.7 months versus 9.7 and 7.0 months, respectively [2, 27]. Especially high-risk patients survived more than twice as long compared to this historical control group. Interestingly, our study included two long-term survivors (i.e. survival ≥ 24 months after diagnosis), both of whom were classified as being high-risk at diagnosis. These two long-term survivors also participated in the first part of our trial, in which they received radiotherapy combined with gemcitabine at diagnosis. Both long-term survivors did not pursue any further treatment after secondary disease progression. Since these patients were not re-irradiated, and the initial treatment was considered not effective based on their PFS (5.1 and 6.3 months, respectively), the prolonged survival could well be a result of the triplet treatment they received in this current trial. Out of nine patients, four were re-irradiated, of whom one received re-irradiation prior to participation in our trial. To what extent additional treatment with re-irradiation has influenced the OS of our trial patients cannot be determined with certainty due to the limited power. However, our study patients that were not re-irradiated showed a survival benefit of 3.6 months, which is comparable to the survival benefit of 3.4 months that may be obtained by re-irradiation [28, 29]. The study patients who did receive re-irradiation, either upfront or after participating in our trial, showed a survival benefit of even 6.2 months. This, together with the radiologic partial response and stable disease observed in four out of nine patients is suggestive of a possible effect of the treatment combination used.

Unfortunately, the response rate of the QoL questionnaires was low in our study. Therefore, it was not possible to assess overall QoL during the treatment period. Patients and their parents who did fill in the questionnaires at two or more time points, reported a stable QoL for most items of the questionnaires during treatment except for (i) physical performance, which is in line with disease course, where patients deteriorate further and loss of neurological functions increases; (ii) nausea, which is the main side effect of chemotherapeutic agents; and (iii) fear of procedures and treatments, caused by anxiety regarding the biweekly procedure of accessing the port-a-cath, and the infusion of bevacizumab and irinotecan. Quality of life research should be made more feasible for this patient population, especially since it is an import tool to assess treatment burden. To do so, shorter and online-provided questionnaires could yield higher response rates and thus more adequate information regarding their experience during and after treatment.

Even though evidence regarding the efficacy of adding bevacizumab and erlotinib to conventional radiotherapy at diagnosis in pediatric HGG and DIPG is limited [30, 31], combining these compounds seems to have at least some potential in DIPG patients. The partial response observed in three patients and stable disease in another patient in this study are promising. Unfortunately, this study had to be terminated prematurely due to logistic difficulties in transferring this study to the new Dutch pediatric oncology center where pediatric oncology care and research are centralized in one specialized pediatric oncology hospital, the Princess Máxima Center. The initial single-center setup of this trial and non-centralized care for DIPG patients in the Netherlands, with an incidence of only nine patients per year [5], resulted in a very slow inclusion rate of (i.e., nine patients in eight years), which was possibly the study's greatest limitation. Centralization of pediatric oncology care and research in the Netherlands is therefore a positive development since now all DIPG patients are treated at one location, which could increase participation rate in future clinical trials in the Netherlands. Besides, we emphasize the need for more international collaborative clinical trials to further increase the inclusion number and rate of such promising trials for DIPG patients in the future.

To conclude, our study demonstrates that administration of daily erlotinib (up to 85mg/m^2^) combined with biweekly bevacizumab (10mg/kg) and irinotecan (125 mg/m^2^) is safe and well tolerated in children with progressive DIPG. Although not powered on efficacy, the median OS of patients treated with this combination is longer than known from literature, especially for high-risk patients. Our findings support the hypothesis that multi-targeted therapy could be of great interest for DIPG patients. Further research is mandatory to determine efficacy of such combinations in larger study populations.

## Supplementary Information

Below is the link to the electronic supplementary material.

Supplementary file1 (PDF 337 kb)

## Data Availability

The datasets generated and analyzed during the current study are available from the corresponding author on reasonable request.
