# Supplementary material for: A phase I/II study of bevacizumab, irinotecan and erlotinib in children with progressive diffuse intrinsic pontine glioma
Source: J Neurooncol. 2021 May 7;153(2):263–71. doi: 10.1007/s11060-021-03763-1 (PMC8211596; doi:10.1007/s11060-021-03763-1)
Supplement: Supplementary file 1 — Supplementary file1 (PDF 337 kb) [file 11060_2021_3763_MOESM1_ESM.pdf]

A phase I/II study of bevacizumab, irinotecan and erlotinib in children with progressive diffuse intrinsic pontine glioma

Running title: Targeted therapy in DIPG

Fatma E. El-Khouly [1,2*], Sophie E.M. Veldhuijzen van Zanten [1,2,3*], Marc H.A. Jansen [1,4], Dewi P. Bakker [5], Esther Sanchez Aliaga [6], N. Harry Hendrikse [6,7], W. Peter Vandertop [8], Dannis G. van Vuurden [1,2#], Gertjan J.L. Kaspers [1,2#]

[1] Emma Children's Hospital, Amsterdam UMC, Vrije Universiteit Amsterdam, Pediatric Oncology, Amsterdam, The Netherlands

[2] Princess Máxima Center for Pediatric Oncology, Utrecht, The Netherlands

[3] Erasmus MC, Radiology & Nuclear Medicine, Rotterdam, The Netherlands

[4] Wilhelmina Children's Hospital, University Medical Center Utrecht, Immunology, Utrecht, the Netherlands

[5] Emma Children's Hospital, Amsterdam UMC, Child Neurology, Amsterdam, The Netherlands

[6] Amsterdam UMC, Vrije Universiteit Amsterdam, Radiology & Nuclear Medicine, Amsterdam, The Netherlands

[7] Amsterdam UMC, Vrije Universiteit Amsterdam, Clinical Pharmacology & Pharmacy, Amsterdam, The Netherlands

[8] Amsterdam UMC, Neurosurgical Center Amsterdam, Amsterdam, the Netherlands

[*] Shared first authorship

[#] Shared last authorship

CORRESPONDING AUTHOR

Fatma E. El-Khouly

Amsterdam UMC, location VUmc - Pediatric Oncology

De Boelelaan 1117, 1081 HV Amsterdam, The Netherlands

Tel.: +31 20 4445056

Email: f.el-khouly@amsterdamumc.nl

Supplementary Table 1: Complete radiologic response assessment of DIPG patients by a neuro-radiologist.

Patient ID	Week	Extension	Pontine involve-ment (%)	T2 hypoin-tensity (%)	Encase-ment (°)	Metas-tasis	Diffusion restriction (%)	Heteroge-neity	Hemorrhage	Enhancement extent (%)	Ring-like enhance-ment	Necrosis (%)	Perfusion	Hyper perfusion extent (%)	Hydro-cephalus	Response (RANO)
1	Diagnosis	sup/post	>67	1-33	181-360	no	1-33	marked	none	67-100	yes	67-100	n.a.	n.a.	yes	n.a.
	Start study	sup	>67	1-33	0-180	no	none	mild	minimal	34-66	yes	34-66	hyper	34-66	no	n.a.
	Week 9	sup	>67	1-33	0-180	no	1-33	marked	minimal	34-66	yes	1-33	hyper	34-66	no	PD
2	Diagnosis	sup/post/inf	>67	none	181-360	no	none	marked	none	1-33	yes	1-33	hyper	34-66	no	n.a.
	Start study	sup/post/inf	>67	1-33	181-360	no	none	marked	none	34-66	yes	1-33	n.a.	n.a.	yes	n.a.
	Week 9	post	51-66	none	none	no	none	mild	minimal	1-33	yes	1-33	normal	n.a.	no	PR
	Week 17	sup/post/inf	>67	none	none	no	none	mild	none	1-33	yes	1-33	normal	n.a.	no	SD
	Week 47	sup/post/inf	51-66	none	none	no	none	mild	none	1-33	yes	1-33	n.a.	n.a.	no	PD
3	Diagnosis	sup/post/inf	>67	1-33	181-360	no	1-33	mild	spotty/minimal	1-33	yes	1-33	normal	n.a.	No	n.a.
	Start study	sup/post/inf	>67	none	0-180	no	none	marked	spotty/minimal	1-33	yes	1-33	n.a.	n.a.	yes	n.a.
	Week 9	sup/post/inf	>67	1-33	181-360	no	1-33	marked	spotty/minimal	1-33	no	none	n.a.	n.a.	yes	PD
4	Diagnosis	post	>67	1-33	181-360	no	n.a.	marked	none	1-33	yes	1-33	n.a.	n.a.	no	n.a.
	Start study	post	>67	1-33	0-180	no	1-33	marked	none	67-100	yes	67-100	n.a.	n.a.	no	n.a.
	Week 9	post	>67	1-33	0-180	no	1-33	marked	none	67-100	yes	1-33	hyper	1-33	no	PR
	Week 17	sup/post	>67	1-33	0-180	no	1-33	marked	spotty/minimal	1-33	yes	1-33	normal	n.a.	no	PD
5	Diagnosis	sup/post/inf	>67	none	181-360	no	none	mild	none	1-33	no	n.a.	hyper	1-33	no	n.a.
	Start study	sup/post/inf	>67	none	181-360	no	1-33	mild	none	34-66	yes	1-33	n.a.	n.a.	no	n.a.
	Week 9	sup/post/inf	>67	none	181-360	no	none	mild	none	1-33	no	none	none	n.a.	no	SD
	Week 17	sup/post/inf	>67	none	181-360	no	none	moderate	none	34-66	no	none	n.a.	n.a.	no	PD
6	Diagnosis	sup/post/inf	100	1-33	full	no	none	mild	no	1-33	no	none	hyper	10	no	n.a.
	Start study	sup/post/inf	100	none	full	no	none	mild	no	1-33	no	none	n.a.	n.a.	yes	n.a.
	Week 9	sup/post/inf	100	none	full	no	n.a.	mild	no	1-33	no	none	n.a.	n.a.	yes	PD
7	Diagnosis	sup/post	>67	34-66	181-360	no	none	marked	yes	1-33	yes	none	n.a.	n.a.	yes	n.a.
	Start study	sup/post/inf	>67	1-33	full	no	none	marked	none	34-66	yes	34-66	n.a.	n.a.	yes	n.a.
	Week 9	sup/post/inf	>67	1-33	full	yes	1-33	marked	none	34-66	yes	34-66	n.a.	n.a.	yes	PD
8	Diagnosis	post	>67	none	181-360	no	none	mild	none	none	no	none	n.a.	n.a.	no	n.a.
	Start study	sup/post/inf	>67	none	181-360	no	none	marked	none	34-66	yes	34-66	n.a.	n.a.	yes	n.a.
	Week 9	post/inf	>67	none	181-360	no	none	moderate	none	1-33	yes	1-33	n.a.	n.a.	no	PR
	Week 17	post/inf	>67	none	181-360	no	none	moderate	none	1-33	yes	1-33	n.a.	n.a.	no	SD
9	Diagnosis	inf	>67	1-33	181-360	no	none	mild	none	none	no	none	n.a.	n.a.	no	n.a.
	Start study	sup/inf	>67	1-33	181-360	no	1-33	mild	none	none	no	none	normal	n.a.	no	n.a.
	Week 9	sup/post/inf	>67	1-33	full	no	none	moderate	none	1-33	no	none	n.a.	n.a.	yes	PD

Sup: superior (midbrain, thalami); post: posterior (cerebellum, brachium pontis); inf: inferior (medulla); PD: progressive disease; PR: partial response; SD: stable disease; n.a.: not applicable
